# Loss of *yata*, a Novel Gene Regulating the Subcellular Localization of APPL, Induces Deterioration of Neural Tissues and Lifespan Shortening

**DOI:** 10.1371/journal.pone.0004466

**Published:** 2009-02-11

**Authors:** Masaki Sone, Atsuko Uchida, Ayumi Komatsu, Emiko Suzuki, Ikue Ibuki, Megumi Asada, Hiroki Shiwaku, Takuya Tamura, Mikio Hoshino, Hitoshi Okazawa, Yo-ichi Nabeshima

**Affiliations:** 1 Medical Top Track Program, Medical Research Institute, Tokyo Medical and Dental University, Tokyo, Japan; 2 Department of Neuropathology, Medical Research Institute, Tokyo Medical and Dental University, Tokyo, Japan; 3 Kyoto University Graduate School of Medicine, Kyoto, Japan; 4 PRESTO, Japan Science and Technology Agency, Kawaguchi, Japan; 5 National Institute of Genetics, Mishima, Japan; Massachusetts Institute of Technology, United States of America

## Abstract

**Background:**

The subcellular localization of membrane and secreted proteins is finely and dynamically regulated through intracellular vesicular trafficking for permitting various biological processes. *Drosophila* Amyloid precursor protein like (APPL) and Hikaru genki (HIG) are examples of proteins that show differential subcellular localization among several developmental stages.

**Methodology/Principal Findings:**

During the study of the localization mechanisms of APPL and HIG, we isolated a novel mutant of the gene, *CG1973*, which we named *yata*. This molecule interacted genetically with *Appl* and is structurally similar to mouse *NTKL/SCYL1*, whose mutation was reported to cause neurodegeneration. *yata* null mutants showed phenotypes that included developmental abnormalities, progressive eye vacuolization, brain volume reduction, and lifespan shortening. Exogenous expression of *Appl* or *hig* in neurons partially rescued the mutant phenotypes of *yata*. Conversely, the phenotypes were exacerbated in double null mutants for *yata* and *Appl*. We also examined the subcellular localization of endogenous APPL and exogenously pulse-induced APPL tagged with FLAG by immunostaining the pupal brain and larval motor neurons in *yata* mutants. Our data revealed that *yata* mutants showed impaired subcellular localization of APPL. Finally, *yata* mutant pupal brains occasionally showed aberrant accumulation of Sec23p, a component of the COPII coat of secretory vesicles traveling from the endoplasmic reticulum (ER) to the Golgi.

**Conclusion/Significance:**

We identified a novel gene, *yata*, which is essential for the normal development and survival of tissues. Loss of *yata* resulted in the progressive deterioration of the nervous system and premature lethality. Our genetic data showed a functional relationship between *yata* and *Appl*. As a candidate mechanism of the abnormalities, we found that *yata* regulates the subcellular localization of APPL and possibly other proteins.

## Introduction

Transmembrane and secreted proteins are synthesized in the ER and are transported to their final destinations, such as the cell surface, by means of vesicular trafficking. This process is tightly regulated both spatially and temporally to establish the proper subcellular distribution of molecules for permitting various biological processes. Such regulation is especially important in neurons that have extremely polarized structures. The regulation of protein trafficking is known to be involved in both normal function and dysfunction of the nervous system.

Some proteins are known to show differential subcellular localization during distinct developmental stages. One such protein is APPL, the functional *Drosophila* homologue of the mammalian Amyloid precursor protein (APP) [1], [2] that is encoded by one of the causative genes in Alzheimer's disease [3]. APPL is a transmembrane protein that is cleaved for secretion [4]. It is transported to synapses at the mid-pupal stage, but it is mainly localized to cell bodies in adults. APPL is suggested to be involved in the formation of larval neuromuscular synapses by regulation of the cell adhesion molecule, Fasciclin II [1], [5], [6]. It also regulates axonal transport, and gene dosages of motor proteins affect the phenotype caused by overexpression of APPL [7], [8]. Other studies have shown that APPL participates in peripheral nervous system development [9], and neurite extension after brain injury [10].

Here, we describe the identification of a novel *Drosophila* gene named *yata* that was fortuitously identified based on its genetic interaction with *Appl*. In addition to APPL, our study also identified HIG and Sec23p as key molecules that are important to the function of YATA. HIG is another protein that shows differential localization to synapses during different developmental stages. Mutation of the *hig* gene results in sluggishness in larvae and adults [11]–[13]. HIG is a member of the immunoglobulin superfamily that is transported and secreted to synaptic clefts from the early stage of pupal synaptogenesis. Its subcellular distribution changes drastically in the pupal brain. At the mid-pupal stage (2–3 days post-pupariation), HIG is transported from cell bodies to synaptic terminals. At the late-pupal stage (4th day post-pupariation), HIG is localized exclusively to cell bodies.

Secretory vesicles have coat structures made up of COPI, COPII and clathrin [14]. Sec23p is a component of the COPII coat of the secretory vesicles that travel from the ER to the Golgi [15], [16]. *Sec23* has been identified as the causative gene of Cranio-lenticulo-sutural dysplasia and related zebrafish mutation [17], [18] that affect the craniofacial chondrocyte maturation. These observations suggest the importance of *Sec23* in the regulation of development.

Here we report phenotypic and functional analyses of *yata*. *yata* mutant flies showed phenotypes including developmental abnormalities, progressive deterioration of the nervous tissues, lifespan shortening, and aberrant accumulation of Sec23p. We also found that an exogenous supply of an excessive dose of *Appl* or *hig* partially rescued the phenotypes of *yata* mutants. Our results suggested that depletion of *yata* profoundly affects the subcellular localization of APPL and possibly other proteins. This may eventually lead to severe deterioration of the nervous system and the premature lethality of animals.

## Results

### Identification of *yata* as a genetically-interacting molecule of *Appl*

While studying the localization mechanisms of APPL and HIG, we made a transgenic fly with an insertion of a P-element containing the HIG minigene in the third chromosome. We crossed these flies with a strain carrying the *Appl^d^* null mutant allele on the X-chromosome [2] to examine interactions between *hig* and *Appl*. Homozygous flies displayed a mild rough eye phenotype; this phenotype was enhanced in the *Appl^d^* hemizygous background, suggesting that the insertional mutation disrupted a gene that interacted with *Appl*. We tried to study the responsible gene and identified a novel gene named ‘*yata*’ after the legendary crow that guided an ancient Japanese emperor, since its gene product was suggested to control protein localization.

**Figure 1:**
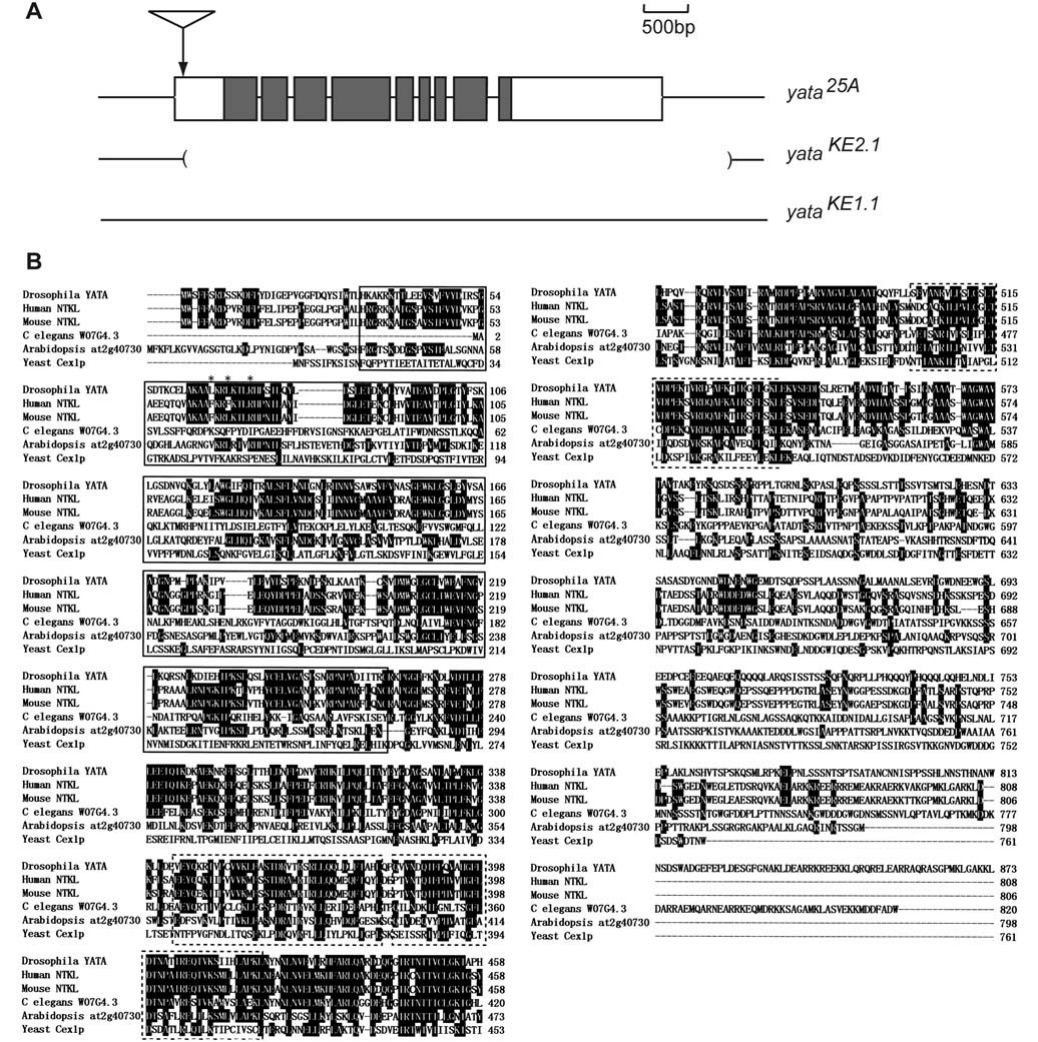
Identification of *yata*. (A) Schematic structure of the *yata* gene. Exons are boxed. Shading indicates putative protein coding regions. Molecular lesions of the *yata* alleles are indicated. The original *yata* mutant was isolated as an insertional allele of a P-element into the first exon (designated as *25A*). We recovered an allele (*KE2.1*), in which almost the entire *yata* gene was deleted along with another allele (*KE1.1*), in which the P-element had been precisely excised. (B) Amino acid alignment of *Drosophila* YATA and its related proteins in other species. Identical residues are highlighted. Protein kinase-like domains are indicated by boxes. HEAT repeats are indicated by broken lines. Conserved amino acids of the binding motif with the AP2 β-appendage ([F/I/L]XX[F/L]XXXR) are indicated by ‘*’. Amino acid sequences are shown for YATA/CG1973 (*Drosophila melanogaster*, AAF56933), NTKL (*Homo sapiens*, AB051427), SCYL1 (*Mus musculus*, AF276514), W07G4.3 (*Caenorhabditis elegans*, NP_506259), AT2G40730 (*Arabidopsis thaliana*, NP_181605) and Cex1p (*Saccharomyces cerevisiae*, NP_014755).

**Figure 2:**
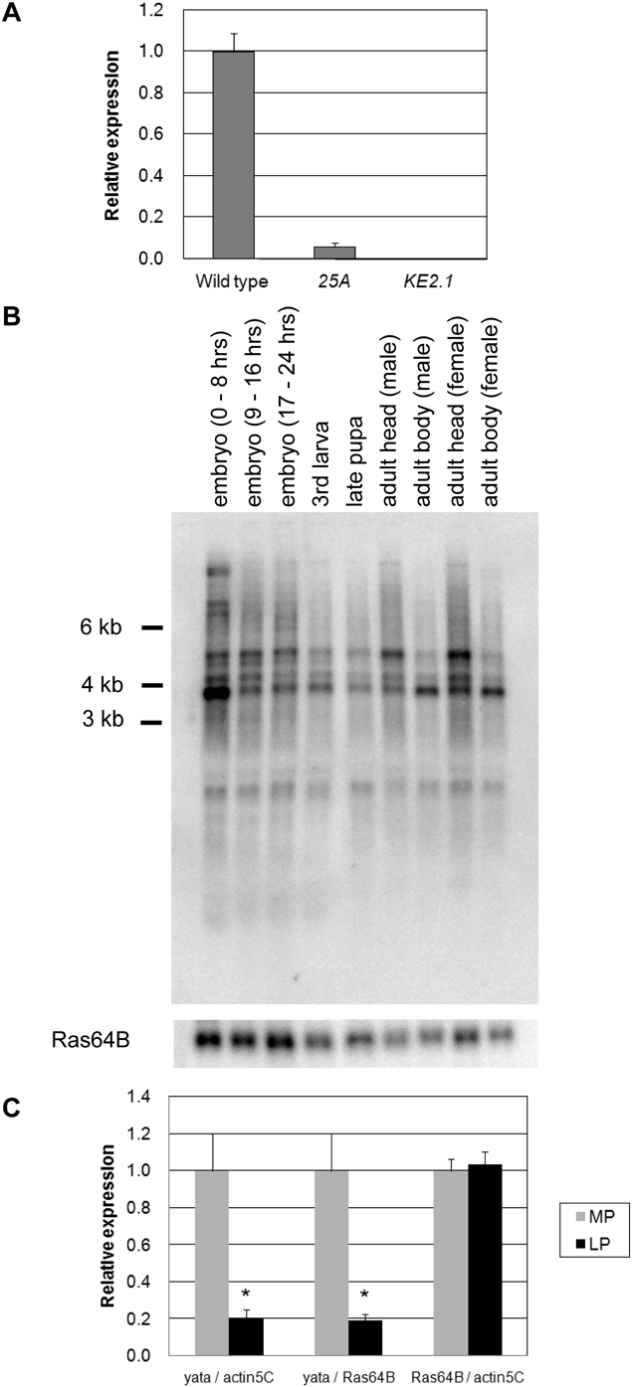
Expression of the *yata* gene. (A) Relative expression of the *yata* gene in the *yata* mutant alleles quantified with real-time quantitative PCR. Expression was reduced to 5% of the wild-type level in *25A* mutants. No transcripts were detected in *KE2.1* mutants. All of the data collected was for whole female adult flies. Numbers of examined samples include 6 for the wild-type, 6 for the *25A* and 5 for the *KE2.1*. Error bars indicate SEM. (B) Developmental Northern analysis of the *yata* gene. Each lane contained 5 µg poly(A)^+^ RNA. (C) Relative expression of the *yata* gene in mid pupae (MP) and late pupae (LP), as quantified with real-time PCR. A significant reduction in the transcript levels was observed in the late pupae compared with *actin5C* and *Ras64B*. All of the data were collected for whole wild-type female pupae. The numbers of samples examined were 5 mid pupae and 4 late pupae. Mid-pupal and late-pupal cDNAs were examined together in single PCR reactions. *: p<0.05 (one-way ANOVA, Welch's test). Error bars indicate SEM.

Genomic Southern analysis and inverse PCR experiments identified the single P-element insertion site within the first exon of the *CG1973* gene (Figure 1A). Quantitative PCR analysis showed that *CG1973* mRNA levels were reduced to approximately 5% of wild-type levels in insertion mutants (Figure 2A). We mobilized the P-element from the original allele (designated as *25A*) using Δ2-3 transposase, and we recovered an allele (*KE1.1*) in which it was precisely excised. In this allele, all of the phenotypes observed in the *CG1973^25A^* homozygotes including abnormal eye morphology and short lifespan were successfully reversed (see below). This finding indicates that *CG1973* was the responsible gene. In the *KE2.1* allele, almost the entire *CG1973/yata* gene was deleted (Figure 1A); no *yata* transcripts were detectable in homozygotes, indicating a null allele (Figure 2A). *yata* cDNAs were amplified by RT-PCR and sequenced, revealing a predicted amino acid sequence identical to AAF56933 in the DDBJ database. This sequence encoded an 873 a.a. protein with an N-terminal protein kinase-like domain (Figure 1B). Several genes of various species, including yeast CEX1p [19] and mammalian NTKL (also referred as SCYL1, Scy1-like1 or CVAK90) [20], [21], show structural similarity to the *Drosophila yata*. *Drosophila* YATA and human NTKL share 46% identity in overall a.a. sequence, although the similarity is low in the C-terminal regions. Both lack conserved a.a. residues in the protein kinase-like domains that are essential for catalytic activity [22]. Recently, *NTKL* was identified as a causative gene in the *mdf* mutant mouse that shows spinocerebellar neurodegeneration [23]. NTKL has also been identified as an interacting molecule of the adaptor protein 2 (AP2) appendage that are involved in clathrin-coated endocytic vesicle formation [24]. The binding motif with the AP2 β-appendage ([F/I/L]XX[F/L]XXXR) is conserved in the *Drosophila* YATA protein.

*yata* was expressed in broad developmental stages/tissues (Figure 2B). Although 3.8 kb transcripts were detected in all of the examined stages/tissues, they were more strongly expressed in the early embryonic stages. Transcripts of different lengths were also detected; these included a 5.4 kb transcript that was abundantly expressed in adult heads. We also performed RT-PCR, 5′ RACE and 3′ RACE analyses. Differences in the cDNA structures were observed in the length of the 3′ UTR, which ranged between 0.6 kb and 1.7 kb. The longest cDNA contained a 1682 bp 3′ UTR that was encoded by a single exon.

### *yata* mutants show progressive deterioration of the nervous system and lifespan shortening

**Figure 3:**
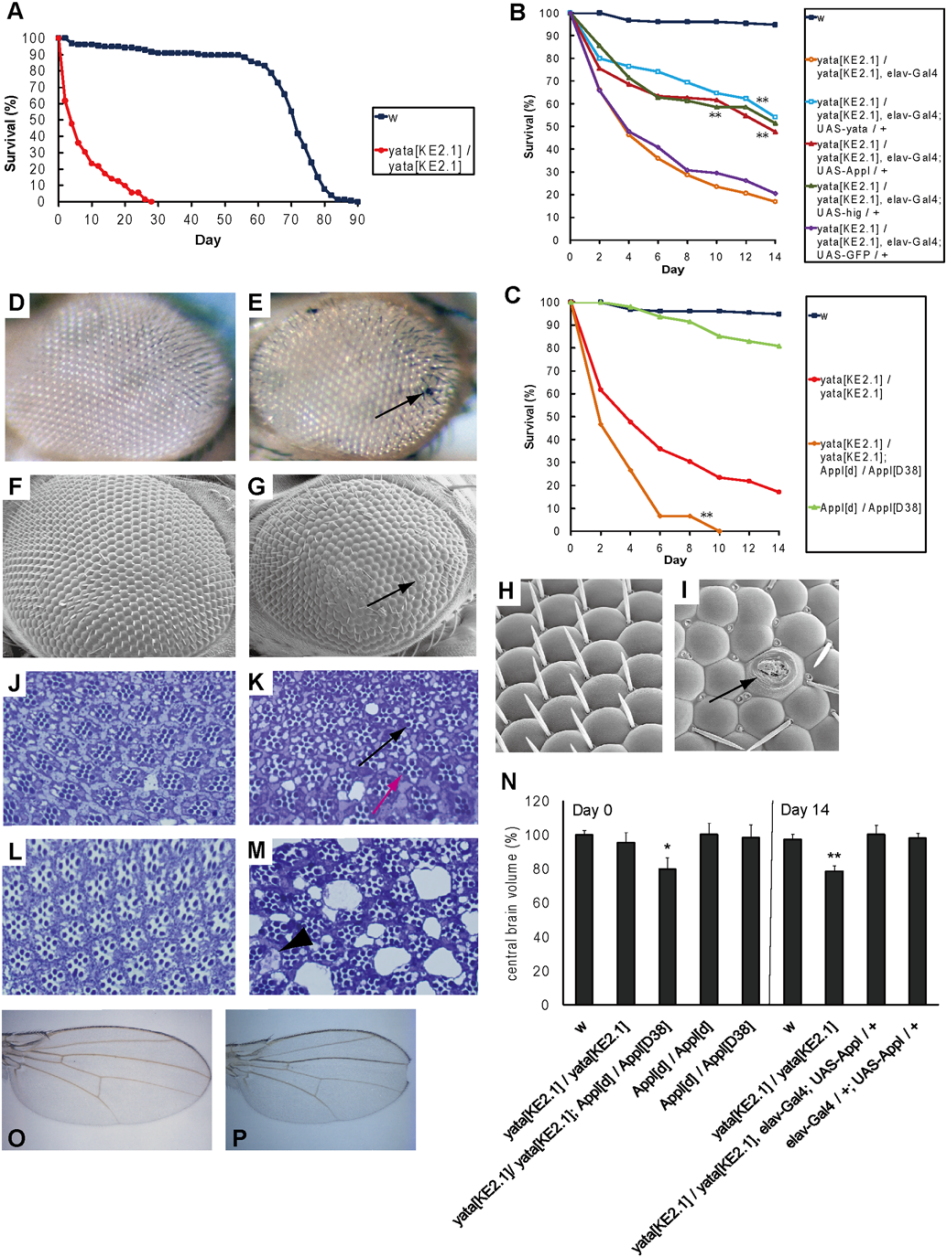
Phenotypes of the *yata* mutant. (A) Lifespan of the *yata* mutants (red, filled circles) compared with *w* (blue, filled squares). (B) Genetic rescue experiment. Survival is shown for *w* (blue, filled squares), *yata* mutants (orange, open circles), rescue by *yata* overexpression (light blue, open squares), rescue by *Appl* (dark red, filled triangles), rescue by *hig* (dark green, open triangles), and rescue by GFP (purple, open diamonds). **: p<0.01 (Log-rank test; compared with *yata* mutants). A partial rescue was noted for the lifespan of *yata^KE2.1^*; *elav-Gal4*; *UAS-yata*, with significant differences found for both *w* (p<0.01) and *yata^KE2.1^* (p<0.01). Similarly, the rescuing effects of APPL or HIG overexpression were significant (p<0.01 and p<0.01, respectively). (C) Genetic interaction between *yata* and *Appl*. Data are shown for *w* (blue, filled squares), *yata* mutants (red, filled circles), *yata*; *Appl* double mutants (orange, filled diamonds), and *Appl* mutants (light green, filled triangles). **: p<0.01 (compared with *yata* mutants). The enhanced effect on the short lifespan phenotype in the *Appl*; *yata* double mutants was significant (p<0.01). The numbers of the examined flies were 154 (*w*), 128 (*w*; *yata^KE2.1^/yata^KE2.1^*), 136 (*w*; *yata^KE2.1^/yata^KE2.1^*, *elav-Gal4*), 85 (*w*; *yata^KE2.1^/yata^KE2.1^*, *elav-Gal4*; *UAS-yata-HA/+*), 115 (*w*; *yata^KE2.1^/yata^KE2.1^*, *elav-Gal4*; *UAS-Appl-HA/+*), 70 (*w*; *yata^KE2.1^/yata^KE2.1^*, *elav-Gal4*; *UAS-hig-HA/+*), 88 (*w*; *yata^KE2.1^/yata^KE2.1^*, *elav-Gal4*; *UAS-GFP/+*), 15 (*yata^KE2.1^/yata^KE2.1^*; *Appl^d^/Appl^D38^*), and 47 (*Appl^d^/Appl^D38^*). (D, E) Compound eye of young *w* (D) and *yata* mutant (E) flies. Black-colored ommatidia were observed in *yata* mutants (arrow in E), in addition to irregular alignments. (F, G) Scanning electron microscopy images of the compound eyes are shown in (D) and (E). A black-colored ommatidium is indicated (arrow) in (G). (H, I) Magnified images of (F) and (G). Abnormal morphology in the outer structure of the lens (arrow in I), bristles and overall ommatidial alignment in *yata* mutants. (J–M) Toluidine blue staining of 1 µm semithin sections of the compound eyes on day 0 *w* (J), day 0 *yata* mutant (K), day 7 *w* (L) and day 7 *yata* mutant (M). Examination in white-eyed background. Tissue polarity defects and abnormal number of rhabdomeres in ommatidia were observed (red and black arrows in K). Increasing numbers of vacuolar structures were observed with increasing age in *yata* mutants (K, M). Vacuoles sometimes contained residual structures (arrowhead in M). (N) Volumes of the central brain were calculated from the measured area and the thickness of the paraffin sections spanning the entire brain. Data were normalized by the average day 0 *w* data. *: p<0.05; **:p<0.01 (one-way ANOVA, Dunnett's test). Error bars indicate SEM. The numbers of the examined samples were 9 (*w*, day0), 5 (*w*; *yata^KE2.1^/yata^KE2.1^*, day0), 5 (*yata^KE2.1^/yata^KE2.1^*; *Appl^d^/Appl^D38^*, day0), 5 (*Appl^d^/Appl^d^*, day0), 6 (*Appl^d^/Appl^D38^*, day0), 10 (*w*, day14), 11 (*w*; *yata^KE2.1^/yata^KE2.1^*, day14), 5 (*w*; *yata^KE2.1^/yata^KE2.1^*, *elavGal4*; *UAS-Appl-HA/+*, day14) and 5 (*w*; *elav-Gal4/+*; *UAS-Appl-HA/+*, day14). (O, P) Wing morphology of the wild type (O) and *yata* mutant (P). All of the data in Figure 3 were collected from female flies of the same genetic background.

*yata^KE2.1^* null mutants displayed premature lethality. Homozygous *yata^KE2.1^* flies started to die after eclosion and exhibited a shorter lifespan than *w* flies that we used as the wild type (Figure 3A). To examine and compare lifespans among different genotypes, all of the chromosomes to be compared were introduced into the same genetic background by consecutive backcrossing for more than three generations. Exogenous induction of *yata* into *yata^KE2.1^* using the neuron-specific *elav-Gal4* driver resulted in a partial but significant rescue (p<0.01; Figure 3B; full lifespan data are shown in Figure S1A), indicating that *yata* expression in neurons is involved in preventing early death. *yata* mutants also displayed various eye defects, including irregular arrangement of ommatidia, black discoloration, and lens defects (Figure 3D–3I). Examination of internal morphology by toluidine blue staining of semithin sections revealed tissue polarity defects, abnormal rhabdomere numbers and vacuolization in *yata* mutants at day 0 after eclosion (Figure 3J, 3K). There was a remarkable increase in the vacuolization at day 7 in the 12 hr light/12 hr dark conditions, suggesting the progressive deterioration of the retinal neural tissue (Figure 3L, 3M). Vacuolization was most severe in the white-eyed mutant retina and less frequently observed in the red-eyed retina and brain. We also examined the light-dependency of the eye vacuolization phenotype. In the constant dark conditions, progressive vacuolization was found to be suppressed (Figure S2). Next we examined the central brain volume of *yata* mutants by measuring the area of the paraffin sections spanning the entire brain. *yata* mutants showed brain volumes indistinguishable from those of wild-type flies at day 0, but these were reduced at day 14 (p<0.01, see Materials and Methods for statistics; Figure 3N). Although its penetrance was low (around 10%), *yata* mutant flies also showed a notched-wing phenotype (Fig. 3P; compare with Fig. 3O).

### *yata* mutant phenotypes were rescued by overexpression of *Appl* and *hig* and exacerbated by the additional ablation of *Appl*

Because there has been a previously observed genetic interaction between *yata^25A^* and *Appl^d^*, we decided to further examine the genetic interaction between *yata^KE2.1^* and *Appl*. We ectopically expressed *Appl* under the control of the neuron-specific *elav-Gal4* driver in the *yata* mutants. Expression of *Appl* in *yata* mutants resulted in a rescue of the early death and progressive reduction of brain volume (Figure 3B, 3N, S1A). Expression of *Appl* in wild-type flies caused no beneficial effects with regard to lifespan or brain volume (Figure 3N, S1B), and the expression of green fluorescent protein (GFP) exhibited no rescue ability (Figure 3B, S1A). In contrast, double null mutants for *yata* and *Appl* showed enhanced early death and reduced brain volume at day 0 (Figure 3C, N). We also examined the lifespan and brain volume of the *Appl* single null mutants. We used two different null alleles, *Appl^d^* and *Appl^D38^* (see Materials and Methods for detailed information). While *Appl^d^/Appl^d^* and *Appl^d^/Appl^D38^* mutants showed similar lifespans, that were shorter than those of the wild-type controls (Figure S1C), their brain volumes were normal at day 0 (Figure 3N). The observed interactions in the brain volume phenotypes indicated a dose-dependent genetic interaction between *yata* and *Appl*, and suggested a functional relationship between these two genes together with the interactions observed in the lifespan phenotypes.

Because APPL and HIG have been suggested to be functionally related, as they control each other's subcellular localization at the late-pupal stage (M.S., unpublished data), we examined the rescuing effect of *hig* overexpression on the *yata* mutant phenotype. Exogenous overexpression of *hig* in *yata* mutant neurons showed a significant ability to rescue the premature death (Figure 3B, S1A), whereas the expression of *hig* itself caused no beneficial effect on lifespan (Figure S1B). These data showed the functional relationship between *yata* and *hig*.

### Subcellular localization of APPL is impaired in *yata* mutants

**Figure 4:**
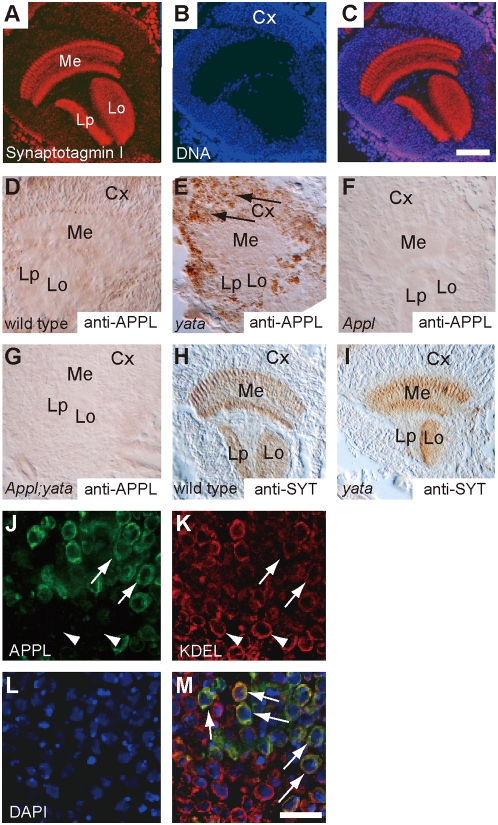
Impaired subcellular localization of endogenous APPL in the *yata* mutant. (A–C) Sections of the mid-pupal brain were stained with anti-Synaptotagmin I antibody (A) and ToPro3 for nucleic acids (B). Anti-Synaptotagmin I antibody labels synaptic neuropils of medulla (Me), lobula (Lo) and lobula plate (Lp), which are surrounded by cortical regions (Cx) that contain cell bodies. Scale bar: 50 µm. (D–G) Sections of the second-day pupal optic lobe were stained by anti-APPL antibody. In the wild type (D), both cortical regions and neuropils were stained; in the *yata* mutant (E), intense staining was observed in the cortical regions (arrows). Both *Appl* (F) and *Appl*; *yata* (G) null mutants showed only background staining. (H, I) Localization of Synaptotagmin I in the wild type (H) and the *yata* mutant (I). Localization of Synaptotagmin I was not changed in the *yata* mutant. (J–M) Localization of endogenous APPL in the *yata* mutant. Immunoreactivity of APPL (J, green) was observed to surround DAPI-positive nuclei (L, blue) and overlap mostly with staining for anti-KDEL antibody (K, red, arrows in M) that labels the ER. In the areas where APPL was intensely accumulated, the immunoreactivity for the anti-KDEL antibody was often reduced (region indicated by arrows in (J, K), compare with the region indicated by arrowheads). Scale bar: 10 µm.

Next, we examined the possible regulatory function of *yata* for *Appl*. The *Drosophila* brain consists of two major parts: cortical regions and neuropils. Cortical regions contain neuronal and a relatively small number of glial cell bodies, whereas neuropils mainly contain synapses [25]. In optic lobe sections, cortical regions (Figure 4B) surround the synaptic neuropils of the medulla, lobula, and lobula plate where Synaptotagmin I is localized (Figure 4A, C) [26]. We raised anti-APPL antiserum and stained the optic lobe sections of second-day pupae. Staining was observed in both cortical regions and neuropils in wild-type pupae (Figure 4D). In *yata* mutants, however, the accumulation of APPL was intensely observed in cortical regions (Figure 4E, arrows). Neither *Appl* single nor *Appl*; *yata* double null mutants showed any significant staining (Figure 4F, 4G). Confocal microscopic analysis revealed a perinuclear localization that mostly overlapped with staining with anti-KDEL antibody (Figure 4J–4M, arrows in Fig. 4M) labeling the ER in *yata* mutants. The accumulation of APPL was observed in a heterogeneous manner in cortical regions. Such accumulation was not observed in the wild-type and *Appl* null mutant brains by the identical procedure of staining and observation (Figure S3). In areas where APPL was intensely accumulated, immunoreactivity for anti-KDEL antibody was often found to be reduced (region indicated by arrows in Fig. 4J, 4K, compared with the region indicated by arrowheads). We performed statistical analysis of the immunostaining intensities of APPL and KDEL in the 10 µm square regions of the single optic lobe and found a significant correlation between them (Figure S4; r = −0.581, Pearson's correlation coefficient, p<0.01).

**Figure 5:**
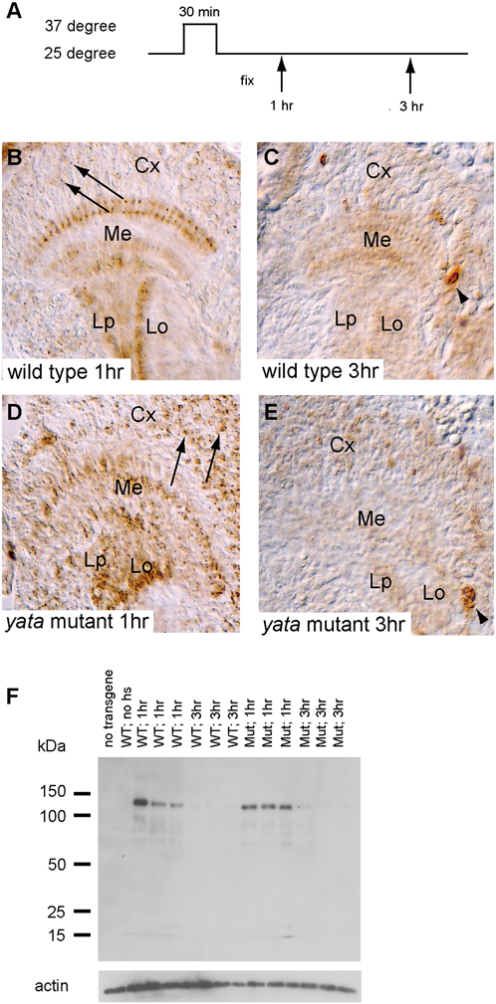
Impaired subcellular localization of exogenously expressed APPL-FLAG in the *yata* mutant brain. (A) Profile for induction using heat shock promoter. (B, C) Localization of APPL-FLAG in mid pupa, fixed at 1 hour (B) and 3 hours (C). (D, E) Localization of APPL-FLAG in the *yata* mutant in mid pupa at 1 hour (D) and 3 hours (E). Punctate signals were observed in cell bodies at 1 hour (arrows in B and D). In *yata* mutants, preferential localization of APPL-FLAG was observed in cell bodies (D, E). A few unidentified cells showed exceptionally strong staining at 3 hours (arrowheads in C and E). Cx: cortical regions, Me: medulla, Lo: lobula, Lp: lobula plate. (F) Western analysis for heat-shock induction of APPL-FLAG using antibody for the C-terminal FLAG tag and anti-actin antibody. Protein lysates of 1/20 portions of single mid pupae were loaded for each lane. At 1 hour after heat shock, the full-length product of APPL-FLAG was induced. This amount decreased at 3 hours in both wild-type and *yata* mutant pupae.

Next, we made a transgenic fly in which expression of APPL conjugated with the C-terminal FLAG tag could be induced by the *hsp70* heat shock promoter. Pupae were heatshocked at 37°C for 30 minutes and examined by FLAG-immunostaining (Figure 5A). In second-day wild-type pupae, APPL-FLAG was expressed in cell bodies as punctuate signals at 1 hour (arrows in Figure 5B) together with the signal already observed in neuropils. Subsequently, it was efficiently transported to neuropils within 3 hours of heat-shock induction (Figure 5C). In *yata* mutants, however, more intense localization of APPL-FLAG was observed in cell bodies at 1 hour (arrows in Figure 5D), and it was preferentially localized to cortical regions even at 3 hours after induction (Figure 5E). The profiles of protein expression/degradation were similar, as revealed by immunoblotting with anti-FLAG antibody (Figure 5F). Full-length APPL-FLAG was induced 1 hour after the heat shock, and the amount decreased at 3 hours.

We also examined subcellular localization of APPL-FLAG in somatic mosaic clones of the *yata^KE2.1^* homozygous cells in the *yata* heterozygous and wild-type cells with the aid of the FLP/FRT system [27]. We induced somatic mosaicism at first instar larvae. Subsequently, second-day pupae were heatshocked to induce APPL-FLAG, and the cortical regions of their optic lobes were examined by FLAG immunostaining at 3 hours after induction. Accumulation of APPL-FLAG was observed in the *yata* mutant clones (Figure S5A–D, surrounded by dotted lines, arrows in Figure S5B), which were denoted by an absence of GFP.

**Figure 6:**
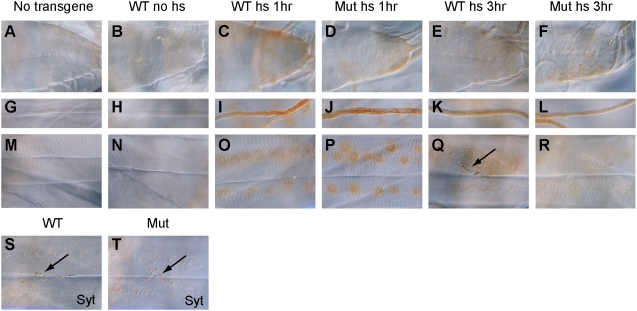
Impaired subcellular localization of exogenously expressed APPL-FLAG in the *yata* mutant motor neurons. (A–R) Third instar larval ventral ganglion (A–F), axons (G–L) and muscles 6 and 7 of the abdominal segments (M–R) were stained with anti-FLAG antibody. Examined larvae were *w* (A, G, M), *w*; *hs-APPL-FLAG/+* (B, H, N: no heat shock; C, I, O; 1 hour; E, K, Q: 3 hours) and *w*; *hs-APPL-FLAG/+*; *yata^KE2.1^/yata^KE2.1^* (D, J, P: 1 hour; F, L, R: 3 hours). In wild-type larva, APPL-FLAG was transported to the neuromuscular synapses at 3 hours after heat shock (arrow in Q). However, in *yata* mutant, synapses were not stained significantly. (R). Synaptic boutons were present in *yata* mutant (arrow in T) similar to the wild type (arrow in S), as was revealed by staining with anti-Synaptotagmin I antibody.

Next we examined subcellular localization of APPL-FLAG in the larval motor neurons. Cell bodies of motor neurons in the ventral ganglion extend their axons, innervate to the body-wall muscles and form neuromuscular junctions as synaptic boutons [28]. Wandering third instar larvae were heatshocked and examined by FLAG immunostaining. Control larvae lacking the transgene showed no FLAG immunoreactivity in the ventral ganglion (Figure 6A), axons (Figure 6G), and neuromuscular junctions on muscles 6 and 7 of the abdominal segments (Figure 6M). *hs-APPL-FLAG* transgenic larvae also showed no staining without heatshock (Figure 6B, H, N). At 1 hour after heat shock, both wild-type and *yata* mutant larvae showed induction of APPL-FLAG in the ventral ganglion, axons and the cell bodies of muscles (Figure 6C, D, I, J, O, P), whereas APPL-FLAG was not localized in the neuromuscular synapses (Figure 6O, P). At 3 hours, wild-type larvae showed localization of APPL-FLAG in the synaptic boutons (arrow in Figure 6Q), in addition to the ventral ganglion and axons (Figure 6E, K). However, in *yata* mutant larvae, APPL-FLAG was not significantly detected in the synaptic boutons (Figure 6R), in contrast to the localization in the ventral ganglion and axons (Figure 6F, L). These results were reproducibly observed in all segments of all examined larvae. On the other hand, synaptic boutons were present in *yata* mutants, as was revealed by immunostaining using anti-Synaptotagmin I antibody (arrows in Figure 6S, T). These data indicate that anterograde delivery of APPL-FLAG to the neuromuscular synapses is impaired in *yata* mutants.

These findings showed that the subcellular localization of APPL is impaired in the *yata* mutants. It is noteworthy that APPL trafficking does not appear to be blocked completely at any specific step, since APPL-FLAG could enter the axons from the cell bodies in the larval motor neurons (Figure 6J, L) and reach the synaptic neuropil in the pupal optic lobe (Figure 5D, E). Thus, the efficiency of one or several steps of APPL trafficking seems to be impaired in *yata* mutants. On the other hand, the synaptic localization of Synaptotagmin I was unchanged in the pupal brain and neuromuscular synapses of *yata* mutants (Figure 4H, I, 6S, T). Therefore, *yata* was suggested to be not a common regulator required for the subcellular localization of ubiquitous proteins but instead a specific regulator of a certain subset of proteins. Our quantitative PCR analysis revealed the downregulation of *yata* expression in late pupae, when synaptic localization of HIG is suppressed (Figure 2C) [11]. This finding suggests a developmental stage-dependent function for *yata*.

### *yata* mutants show aberrant accumulation of Sec23p

**Figure 7:**
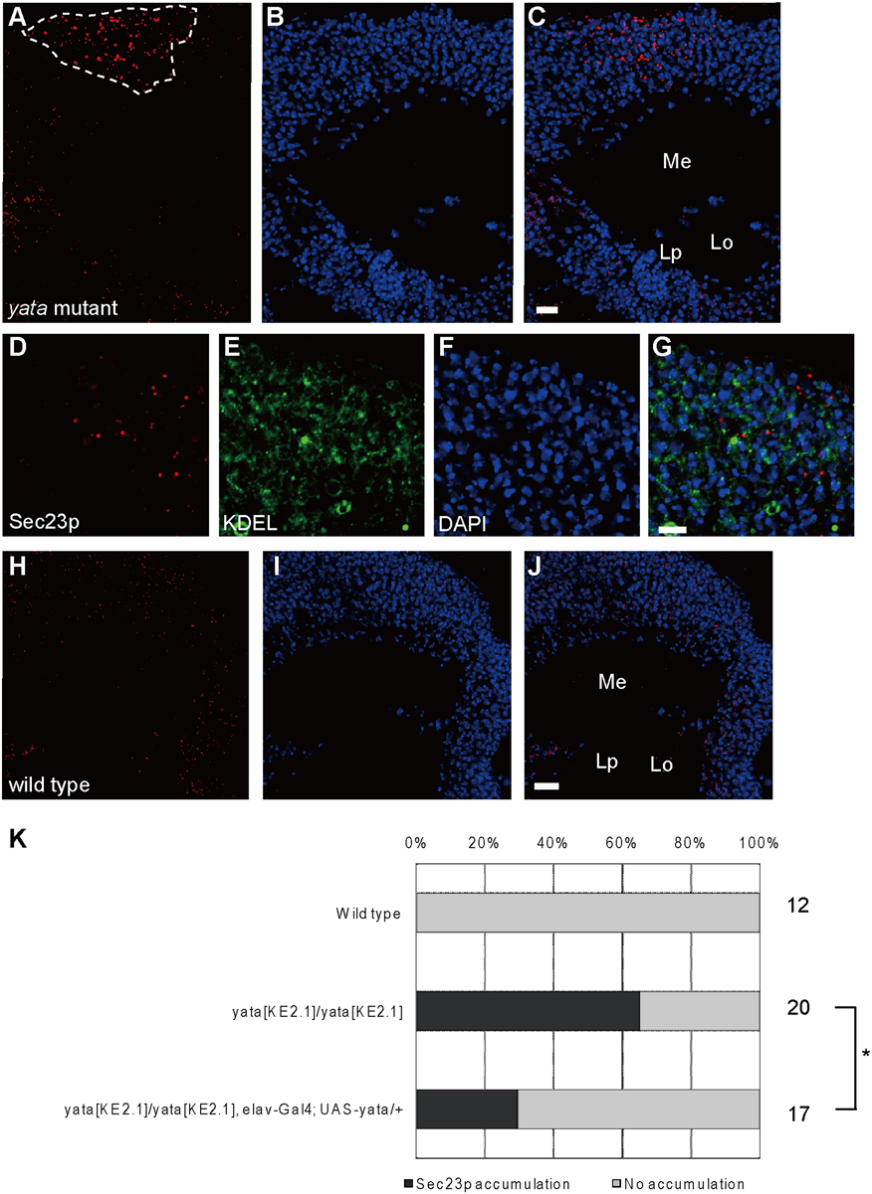
Aberrant accumulation of Sec23p in the *yata* mutant. (A–C) Mid pupal brain of the *yata* mutant stained with anti-Sec23p (A) and DAPI (B). Aberrant accumulation of Sec23p was observed in a restricted region (surrounded by dotted line in A). (D–G) Magnified image stained for anti-Sec23p (D), anti-KDEL (E) and DAPI (F). (H–J) Wild-type brain stained with anti-Sec23p (H) and DAPI (I). Me: medulla, Lo: lobula, Lp: lobula plate. Scale bars: 10 µm in (C) and (J), 5 µm in (G). (K) Summary of Sec23p accumulation phenotype. Ratios of the number of optic lobes that showed the accumulation of Sec23p are shown. The numbers of examined optic lobes are also indicated. *: p<0.05 (chi-square test). All of the data were collected from flies of the same genetic background.

Sec23p is a component of the COPII coat of the secretory vesicles traveling from the ER to the Golgi [15], [16]. While anti-Sec23p staining revealed a punctuate localization that preferentially labeled the ER exit sites (as previously shown by immunoelectron microscopy [29], [30]) (Figure 6H–6J), we found that aberrant accumulation of Sec23p was occasionally observed in a restricted region of the mid-pupal optic lobe of *yata* mutants (Fig. 7A–7C). Accumulated signals were located in perinuclear regions (Figure 7D–7G). Interestingly, such accumulation was consistently observed in the partial region of the optic lobe cortex (surrounded by the dotted line in Figure 7A). The brain regions where such a phenotype was observed differed between individual animals, although their sizes were typically similar. This phenotype was always drastic and could be distinguished easily. Intensity profiles of Sec23p immunostaining showed radically elevated Sec23p signals in the specific region (Figure S6A, B; blue arrow in Figure S6A). We examined 20 *yata^KE2.1^* mutant optic lobes, and similar phenotypes were observed in 13 of them. These phenotypes were not observed in the 12 wild-type optic lobes examined (Figure 7K). This phenotype was less frequently observed in the pupae rescued by the neuronal expression of *yata* (p<0.05, chi-square test). These data highlight the importance of *yata* in the regulation of trafficking from the ER to the Golgi.

## Discussion

Here, we report the identification of the novel gene, *yata*. Null mutants of *yata* showed morphological abnormalities of the eye and wing, early-onset and progressive deterioration of eye and brain, and a shortened lifespan. Our genetic data showed a functional relationship between *yata* and *Appl*. Our data also revealed the molecular function of *yata* in the regulation of the subcellular localization of APPL.

The premature lethality of *yata* mutants was partially but definitely rescued by the neuronal expression of *yata*. These data suggest that the mechanisms of protein localization mediated by neuronal YATA are essential for the homeostasis of the nervous system. Because the expression of *yata* was observed in the all of the examined developmental stages or tissues, its function in other tissues may also contribute to preventing premature lethality.

The mammalian NTKL (also referred as SCYL1, Scy1-like1 or CVAK90) is structurally similar to YATA. NTKL is implicated in clathrin-coated endocytic vesicle formation [24]. Recently, NTKL was also found to be involved in the COPI-mediated retrograde trafficking from the Golgi to the ER [31]. Together with our data, these findings suggest that the regulation of protein localization is the evolutionally-conserved function of YATA and its related proteins in mammals, although the functional similarity between *Drosophila* YATA and mammalian NTKL remains to be further elucidated. Moreover, *NTKL* has been identified as a causative gene in the *mdf* mutant mouse that shows neurodegeneration [23]. These lines of evidence suggests the importance of the *yata*- or *NTKL*-dependent regulation of protein localization as the evolutionally-conserved mechanisms that are required for the neural homeostasis.

Our study revealed that subcellular localization of APPL is impaired in the pupal optic lobes and larval motor neurons. In the *yata* mutant brain, APPL was strongly accumulated in cell bodies. This accumulation was observed in perinuclear regions in many cells. Accumulation of APPL and reduced immunoreactivity of the anti-KDEL antibody showed a significant correlation. Thus the impaired trafficking of APPL and possibly other proteins may lead to dysfunction of basic cellular physiology or architecture, which might eventually cause cell death and tissue deterioration. Another observed cellular dysfunction of *yata* mutants is the accumulation of Sec23p, which was occasionally observed in the restricted regions of the pupal optic lobe. The reason why only restricted regions were affected is unclear and remains to be elucidated. It is noteworthy that *yata* mutant optic lobes show reduced volumes at day 0 (M. S., unpublished data), while central brain volumes are normal at day 0. Therefore, the accumulation of Sec23p may be related to the cellular dysfunction caused by the *yata* mutation, which may eventually result in cell death.

*yata* mutants showed progressive vacuolization in the white-eyed retina, although it is not known if the retinal pathologies of *yata* mutants are caused by similar mechanisms to the brain. We examined the light-dependency of the eye vacuolization phenotype. In the constant dark condition, progressive vacuolization was found to be suppressed. These data suggest that active phototransduction is necessary for the development of progressive vacuolization in *yata* mutants. Mechanisms of phototransduction might participate in the degeneration of retinal cells, similarly to the previously identified *Drosophila* mutants that show light-induced retinal degeneration [32]. Alternatively, synaptic activity of the photoreceptor neurons might be involved. Further investigation is required together with examination of the involvement of APPL in the retinal phenotypes of *yata* mutants.

Our data identified APPL as the functional target molecule of YATA. Although *Appl* null mutant flies are viable and do not show drastic phenotypes [2], *yata* deficiency phenotypes were more severely deteriorated by the additional ablation of *Appl*. The double mutants null for *yata* and *Appl* showed reduced brain volume at day 0 after eclosion, whereas both single mutants exhibited normal volumes at day 0. Conversely, the premature lethality and brain volume reduction at day 14 were improved by the neuronal overexpression of *Appl*. Although overexpression of *Appl* is reported to interfere with axonal transport and some aspects of the nervous system [7], [8], [33], severe detrimental effects were not observed in our experiments; this may be due to the fact we used the *elav* promoter, which is known to be weak. Thus, our brain volume data indicate a dose-dependent genetic interaction between *yata* and *Appl*, and they suggest a functional relationship between these two genes, together with interactions that were observed in the lifespan phenotypes. It is noteworthy that a neuroprotective function of *Appl* has been previously suggested by the phenotype of the double mutant for *Appl* and *löchrig* [34]. On the other hand, the rescue of premature lethality was only partial. Because expression of *hig* also partially rescued the short lifespan phenotype of *yata*, and the subcellular localization of HIG was weakly affected in *yata* mutants (M. S., unpublished data), HIG and other unidentified target molecules of *yata* may also contribute to the mutant phenotypes caused by *yata*.

APPL is a homologue of mammalian Amyloid precursor protein (APP), which is one of the identified causative genes of familial Alzheimer's disease [3]. NTKL has also been identified as a component of ‘ataxiome’, which is a network of interacting proteins comprised of inherited ataxia-inducing molecules [35]. Therefore, further functional analyses of *NTKL* may reveal its possible relationship to neurodegenerative diseases.

NTKL is implicated in both clathrin- and COPI-coated vesicle trafficking [24], [31]. On the other hand, mutation of *Drosophila yata* caused the accumulation of APPL in cell bodies, and this accumulation often overlapped with an ER marker. The accumulation of Sec23p, which is a component of the COPII coat of anterograde secretory vesicles traveling from the ER to the Golgi, was also observed in *yata* mutants. Yeast Cex1p is involved in the nuclear export of tRNA [19]. Mouse NTKL is enriched in synapses [23], and the preferential localization of human NTKL in centrosomes has been also observed [20]. Therefore, YATA and its structurally-related proteins in other species might have different cellular functions at distinct subcellular sites. Alternatively, YATA and its related proteins might have multiple cellular functions at several subcellular locations. Further analysis to identify the functional subcellular site of *Drosophila* YATA will be required.

The subcellular localization of APPL and HIG is regulated dynamically during different developmental stages [1], [11]. In late pupae, the expression of *yata* is downregulated when trafficking of HIG is suppressed. On the other hand, the localization of Synaptotagmin I, which is constantly efficiently transported to synapses [26] (M. S., unpublished data), was not affected in *yata* mutants. These findings raise the possibility that *yata* is involved in the conditional regulation of trafficking of a certain subset of proteins. Further identification of the target and interacting molecules of *yata* would clarify the specialized cellular mechanisms that are involved in the regulation of protein localization as well as their relevance to neuronal survival and possibly to neurodegenerative diseases.

## Materials and Methods

### Fly genetics

Flies were reared at 25°C with a 12 hours light/12 hours dark cycle. Transgenic flies were created by P-element mediated transformation using standard techniques. Transgenic flies used in this study were *UAS-hig-HA*, *hs-Appl-FLAG*, *UAS-Appl-HA* and *UAS-yata-HA*.

### *Appl* mutant alleles

*Appl^d^* fly was a gift from Dr. Kalpana White. The *Appl^d^* allele was originally isolated by engineering chromosomal aberrations, resulting in deletion of the central region of the *Appl* gene, while retaining both its 5′ and 3′ ends [2]. In *Appl^D38^* flies, *Appl* and the adjacent *vnd* gene are deleted [36]. Thus, both are the null alleles of the *Appl* gene, although the *vnd* gene is additionally disrupted in *D38*. Our quantitative RT-PCR analysis failed to detect *Appl* transcript in the *Appl^d^/Appl^d^* and *Appl^d^/Appl^D38^* flies, confirming the null mutation. These two combinations of alleles showed similar phenotypes in our experiments of lifespan and brain volume phenotypes.

### Cloning of the *yata* gene

We extracted poly(A)^+^ RNA from female adult heads using QuickPrep mRNA purification kit (Amersham), and synthesized cDNA using Marathon cDNA amplification kit (Clontech). Subsequently, we performed RT-PCR, 5′ RACE and 3′ RACE analyses. The 5′ ends of the identified cDNAs were located at nucleotide 15290 of the genome sequence recorded as AE003771. In the *yata^25A^* allele, a P-element containing the heat shock promoter and *hig-HA* minigene was inserted 44 bp from the beginning of the first exon. In the *yata^KE2.1^* allele, the 6148 bp genomic region from the 15342 to 21490 position was deleted and a 10 bp sequence of the 3′ end of the P-element then inserted. This was revealed by sequencing the PCR product from *yata^KE2.1^* genomic DNA.

### Plasmid constructs

A combination of polymerase chain reaction, synthetic oligonucleotide and restriction enzyme digestion methods was used in order to generate plasmid constructs. The construct of *hig* was made from type 3 cDNA (DDBJ accession number: D13886). For *hig-HA*, one threonine and three consecutive repeats of the HA tag sequence (YPYDVPDYA) were added to the C-terminus. This was then cloned into the pUAST vector. For *Appl-FLAG and Appl-HA*, the EST clone encoding the full-length APPL protein (clone GH04413) was used. After the entire coding region was subcloned into pBluescriptII SK^−^, DNA sequences encoding three consecutive repeats of the FLAG tag (DYKDDDDK) or HA tag were inserted just before the stop codon. The construct was then cloned into the pCaSpeRhs and pUAST vectors. For the entire coding region of the *yata* gene, DNA sequences encoding one lysine and three consecutive repeats of the HA tag were inserted just before the stop codon. The construct was then cloned into pUAST.

### Northern analysis

Northern analysis was performed as previously described [12].

### Quantitative PCR

Pupae on the second day after pupariation were collected as mid pupae. Pupae with black wings were collected as late pupae. Total RNA was prepared from whole adult flies or pupae using RNAeasy Mini (Qiagen). Each RNA sample was prepared from 5 to 15 flies or pupae. cDNA was synthesized with random primers using First strand cDNA synthesis kit (GE). Quantitative PCR analyses were performed with the 7300 Real-Time PCR System (Applied Biosystems) using the Taqman probe for *yata* (for the sequences in the fifth exon, forward primer: TCGCAGCCAAAGTGATTCG, reverse primer: CGCTGGCTTGGACAGATTG, Taqman probe: CTCGACCACGACCGCCTTTAACTGG), the Taqman probe for *Appl* (for the sequences in the sixth exon, forward primer: CCAAGGCAGCCCAGTCATT, reverse primer: TCTTCCTCGAGTGCCTGAACA, Taqman probe: ATGACGGCTCGCTTCCAGACTT) and *Ras64B* (for exon boundary 2 to 3). For the control, the Taqman probe for *actin5C* (forward primer: CCGAGCGCGGTTACTCTTT, reverse primer: CAACATAGCACAGCTTCTCCTTGAT, Taqman probe: CCGCTGAGCGTGAAATCGTCCGT) was used.

### Anti-APPL antiserum

Anti-APPL antiserum was raised against a fusion protein that consisted of the extracellular portion of the APPL protein (a.a. 111 to 378) and Glutathione-S-transferase derived from the pGEX4T-1 vector (GE). After the fusion protein was expressed in the *E. coli* strain, BL21, it was purified using Bugbuster GST bind purification kit (Takarabio). Then it was injected into rabbits for immunization.

### Immunohistochemistry of the pupal brain

Pupae on the second day after pupariation were collected as mid pupae. The pupal case was removed and the whole pupa was fixed. Samples were fixed in 4% paraformaldehyde in phosphate-buffered saline (PBS) for 1.5 hours at room temperature (RT) followed by successive incubations in 5% and 10% sucrose for 30 minutes at RT, 15% and 20% sucrose for 1 hour at RT, and 30% sucrose overnight at 4°C. All sucrose solutions were in PBS. After being frozen in OCT compound (Tissue Tek) in dry ice/n-hexane, the heads were cut into 10 µm sections using a cryostat microtome. Sections were stained with antibodies using the ABC elite system (Vector). The antibodies used were mouse affinity-purified anti-FLAG M2 (Sigma, diluted 1∶1000), rabbit anti-Synaptotagmin I (diluted 1∶500), rabbit anti-APPL purified with the Hitrap protein A column (GE; diluted 1∶500) and biotin-conjugated secondary antibodies (Vector). Stained sections were dehydrated with a series of ethanol, treated with xylene and mounted with Enteran Neu (Merck). Samples were observed with Axioskop (Zeiss). Photos were taken using MC200 (Zeiss), then scanned and processed using Photoshop CS2 (Adobe). Only brightness and contrast were adjusted for the entire images. For confocal microscopy, sections were stained with mouse anti-KDEL (Stressgen, diluted 1∶200), rabbit anti-GFP (Molecular Probes; diluted 1∶1000), Alexa-488 (Molecular Probes) and Cy3 or Cy5-conjugated (Jackson) secondary antibodies, ToPro3 (Molecular Probes, diluted 1∶2500) and DAPI (Wako Pure Chemicals, Japan, diluted 1∶5000). Samples were then observed with LSM510meta (Zeiss). Obtained images were then processed using LSM image browser (Zeiss) and Photoshop CS2 (Adobe). Statistical analysis of the signal intensity was performed by SPSS 16.0.

### Immunohistochemistory of larval motor neurons

Wandering third instar larvae were dissected, fixed and stained as previously described [13].

### Examination of Sec23p accumulation

Second-day pupal brains were stained with rabbit anti-dSec23p (Affinity Bioreagent, diluted 1∶200), Cy3-conjugated secondary antibody (Jackson), and DAPI. All of the stained sections were examined for the occurrence of aberrant accumulation of Sec23p. This phenotype was always drastic and could be distinguished with no ambiguity. In many cases, both sides of the optic lobes were examined from a pupa.

### Mosaic analysis

Mosaicism was induced by heat shock treatment of first instar larvae at 38°C for 1 hour using *hs-flp* and *FRT82B*. Mosaic clones were distinguished by the absence of GFP immunoreactivity derived from *Ubi-GFP*.

### Morphological analysis of the compound eye

For the analysis of the semithin sections of the compound eye, the specimens were processed using a standard technique that has been previously described [37]. The number of vacuoles that had a longest diameter of over 5 µm was counted. Vacuoles that contained residual cellular structures were omitted from counting. R7-level retinas were observed, and only a vacuole whose entire structure was located in the R7-level was counted. To examine light-dependency, *yata* mutant flies were examined in the constant dark conditions, with the exception of light exposure during dissection (less than 5 minutes).

### Western analysis

Whole pupae were homogenized in SDS-PAGE sample buffer (62.5 mM Tris-HCl, pH 6.8, 2% SDS, 2.5% 2-mercaptoethanol, 5% glycerol, and 0.0025% bromphenol blue), boiled, and electrophoresed on SDS polyacrylamide gels. Protein lysates of 1/20 portions of a single pupa were loaded for each lane. Western analysis was performed based on a previously described method [38], using an Immobilon-P transfer membrane (Millipore), affinity-purified anti-FLAG M2 antibody (Sigma, diluted 1∶500), and anti-actin MAb1501R (Chemicon, diluted 1∶1500).

### Measurement of lifespan

All of the chromosomes were introduced into the same genetic background by consecutive backcrossing for more than three generations. In a standard plastic vial, 1 to 20 flies were reared at 25°C degree. Statistic analysis of lifespan data was performed with the log-rank test using SPSS 16.0.

### Measurement of brain volume

For the measurement of brain volume, adult heads were collected, the probosces were removed, and the samples were fixed in 4% paraformaldehyde in PBS overnight. The samples were then rinsed with PBS for 15 minutes and dehydrated with 70% and 90% ethanol for 15 minutes, 35% n-butanol, 50% ethanol, and 15% water for 15 minutes, 55% n-butanol, 40% ethanol, and 5% water for 15 minutes, 75% n-butanol, and 25% ethanol for 15 minutes, and then 100% n-butanol for 15 minutes. Subsequently, samples were embedded in paraffin, and 6 µm frontal sections prepared from the entire brain were stained with hematoxylin-eosin. Images were obtained from all of the sections using Metamorph ver 7.1.2.0 (Molecular Devices). Then the volume of the central brain was measured by a planimetric method with the aid of Photoshop CS2 (Adobe), via comparison with the image of an objective micrometer. Central brain and optic lobe regions were distinguished by their differential staining reactivity for hematoxylin. Statistic analysis was performed using SPSS 16.0. A significant reduction of brain volume was observed (p<0.01, one-way ANOVA) for the day 0 *yata*; *Appl* double mutant (p<0.05, Dunnett's test) and the day14 *yata* mutant (p<0.01) in comparison with the day0 *w*.

### Scanning electron microscopy analyses

For scanning electron microscopy analyses, specimens were dehydrated in 30%, 50%, and 70% acetone in water for 5 minutes, 90% acetone for 10 minutes, and 99.9% acetone three times for 10 minutes. They were then dried under a vacuum, coated with gold/palladium, and observed with Hitachi S-4700 scanning electron microscope.

## Supporting Information

Figure S1 Lifespan of control genotypes. (A) Full lifespan data for the genetic rescue experiment shown in Figure 3B. Survival is shown for *w* (blue, filled squares), *yata* mutants (pink, filled circles and orange, open circles), rescue by *yata* overexpression (light blue, open squares), rescue by *Appl* (dark red, filled triangles), rescue by *hig* (dark green, open triangles), and rescue by GFP (purple, open diamonds). (B) Survival is shown for *w* (blue, filled squares), *elav-Gal4/+* flies (light blue, filled diamonds), *elav-Gal4/+*; *UAS-Appl/+* flies (orange, filled circles), and *elav-Gal4/+*; *UAS-hig/+* flies (dark red, filled triangles). Overexpression of *Appl* or *hig* resulted in a slightly shortened life span. (C) Survival is shown for *w* (blue, filled squares), *Appl^d^/Appl^D38^* flies (red, filled circles) and *Appl^d^/Appl^d^* flies (orange, filled triangles). *Appl* null mutants with two different genotypes showed similar lifespans, shorter than those of the *w* control flies from the same genetic background. The numbers of examined flies were 154 (*w*), 128 (*w*; *yata^KE2.1^/yata^KE2.1^*), 136 (*w*; *yata^KE2.1^/yata^KE2.1^*, *elav-Gal4*), 85 (*w*; *yata^KE2.1^/yata^KE2.1^*, *elav-Gal4*; *UAS-yata-HA/+*), 115 (*w*; *yata^KE2.1^/yata^KE2.1^*, *elav-Gal4*; *UAS-Appl-HA/+*), 70 (*w*; *yata^KE2.1^/yata^KE2.1^*, *elav-Gal4*; *UAS-hig-HA/+*), 88 (*w*; *yata^KE2.1^/yata^KE2.1^*, *elav-Gal4*; *UAS-GFP/+*), 11 (*yata^KE2.1^/yata^KE2.1^*; *Appl^d^/Appl^D38^*), 47 (*Appl^d^/Appl^D38^*), 92 (*w*; *elav-Gal4/+*), 92 (*w*; *elav-Gal4/+*; *UAS-Appl-HA/+*), 39 (*w*; *elav-Gal4/+*; *UAS-hig-HA/+*) and 44 (*Appl^d^/Appl^d^*). All of the data in Figure S1 were collected from female flies introduced into the same genetic background.
(0.59 MB TIF)

Figure S2 Measurement of vacuolization in the compound eye. The number of vacuoles with a longest diameter of over 5 µm was measured in the compound eyes of different genotypes, of different ages and under different light-conditions. An increased number of vacuoles was observed in day 7 *yata* mutants, although this number was suppressed in constant dark conditions. The numbers of the examined samples were 2 (*w*, day0), 4 (*w*, day7), 2 (*w*; *yata^KE2.1^/yata^KE2.1^*, day0), 5 (*w*; *yata^KE2.1^/yata^KE2.1^*, day7), 4 (*w*; *yata^KE2.1^/yata^KE2.1^*, day7, constant dark),
(2.52 MB TIF)

Figure S3 Confocal microscopic analyses of APPL localization. Localization of endogenous APPL in the wild-type, *yata* null mutant and *Appl* null mutant pupae. The pupae were examined and observed under identical conditions. Immunoreactivity of APPL was observed to accumulate in cell bodies of *yata* mutant pupa (E), but not in wild-type (A) and *Appl* mutant pupae (I). Staining with the anti-KDEL antibody (B, F, J), which labels the ER; DAPI, which labels nuclei (C, G, K); and merged images (D, H, L) are also shown. Scale bars: 5 µm.
(2.57 MB TIF)

Figure S4 Correlation of the signal intensities of APPL and KDEL immunostaining. Statistical analysis of the immunostaining intensities of APPL and KDEL in the 10 µm square regions of the single optic lobe. A significant correlation was observed between the staining intensities of these two antibodies (r = −0.581, Pearson's correlation coefficient, p<0.01, N = 30).
(0.71 MB TIF)

Figure S5 Genetic mosaic analyses of the subcellular localization of exogenously expressed APPL-FLAG. Accumulation of APPL-FLAG was observed in the *yata* mutant clones (A–D, surrounded by dotted lines, arrows in B), which were marked by the absence of GFP (A). Scale bar: 5 µm.
(2.26 MB TIF)

Figure S6 Profiles of Sec23p immunostaining. (A) Intensity profiles of Sec23p immunostaining of the *yata* mutant shown in Figure 7A. Radically elevated Sec23p signals are shown in the indicated region (blue arrow). (B) Such strong signals were not observed in the wild-type pupae, see Figure 7H.
(0.37 MB TIF)
