# Supplementary figures and images for: Loss of yata, a Novel Gene Regulating the Subcellular Localization of APPL, Induces Deterioration of Neural Tissues and Lifespan Shortening
Source: PLoS One. 2009 Feb 11;4(2):e4466. doi: 10.1371/journal.pone.0004466 (PMC2635962; doi:10.1371/journal.pone.0004466)

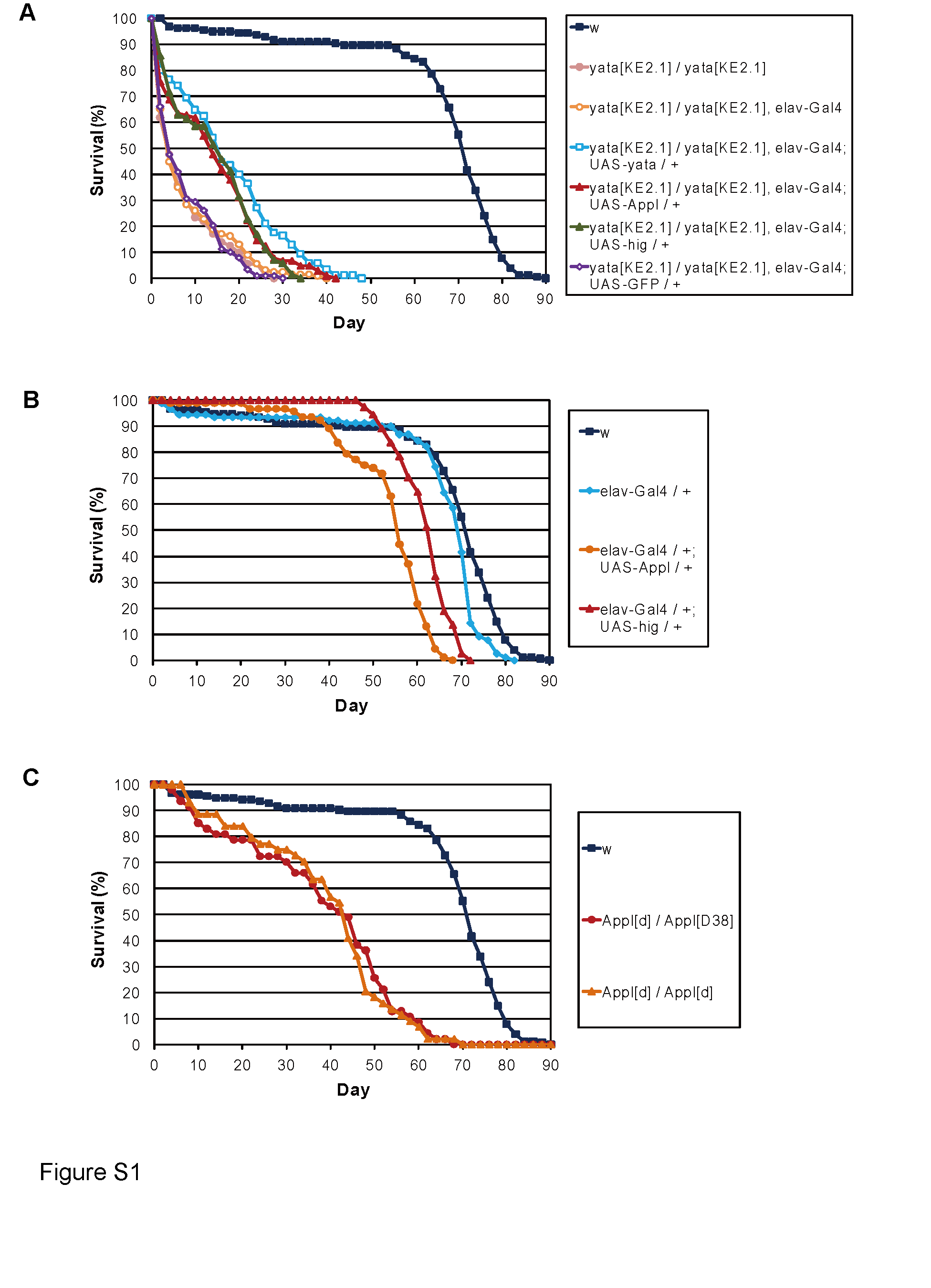

Supplement: Figure S1 — Lifespan of control genotypes. (A) Full lifespan data for the genetic rescue experiment shown in Figure 3B. Survival is shown for w (blue, filled squares), yata mutants (pink, filled circles and orange, open circles), rescue by yata overexpression (light blue, open squares), rescue by Appl (dark red, filled triangles), rescue by hig (dark green, open triangles), and rescue by GFP (purple, open diamonds). (B) Survival is shown for w (blue, filled squares), elav-Gal4/+ flies (light blue, filled diamonds), elav-Gal4/+; UAS-Appl/+ flies (orange, filled circles), and elav-Gal4/+; UAS-hig/+ flies (dark red, filled triangles). Overexpression of Appl or hig resulted in a slightly shortened life span. (C) Survival is shown for w (blue, filled squares), Appld/ApplD38 flies (red, filled circles) and Appld/Appld flies (orange, filled triangles). Appl null mutants with two different genotypes showed similar lifespans, shorter than those of the w control flies from the same genetic background. The numbers of examined flies were 154 (w), 128 (w; yataKE2.1/yataKE2.1), 136 (w; yataKE2.1/yataKE2.1, elav-Gal4), 85 (w; yataKE2.1/yataKE2.1, elav-Gal4; UAS-yata-HA/+), 115 (w; yataKE2.1/yataKE2.1, elav-Gal4; UAS-Appl-HA/+), 70 (w; yataKE2.1/yataKE2.1, elav-Gal4; UAS-hig-HA/+), 88 (w; yataKE2.1/yataKE2.1, elav-Gal4; UAS-GFP/+), 11 (yataKE2.1/yataKE2.1; Appld/ApplD38), 47 (Appld/ApplD38), 92 (w; elav-Gal4/+), 92 (w; elav-Gal4/+; UAS-Appl-HA/+), 39 (w; elav-Gal4/+; UAS-hig-HA/+) and 44 (Appld/Appld). All of the data in Figure S1 were collected from female flies introduced into the same genetic background. (0.59 MB TIF) [file pone.0004466.s001.tif]

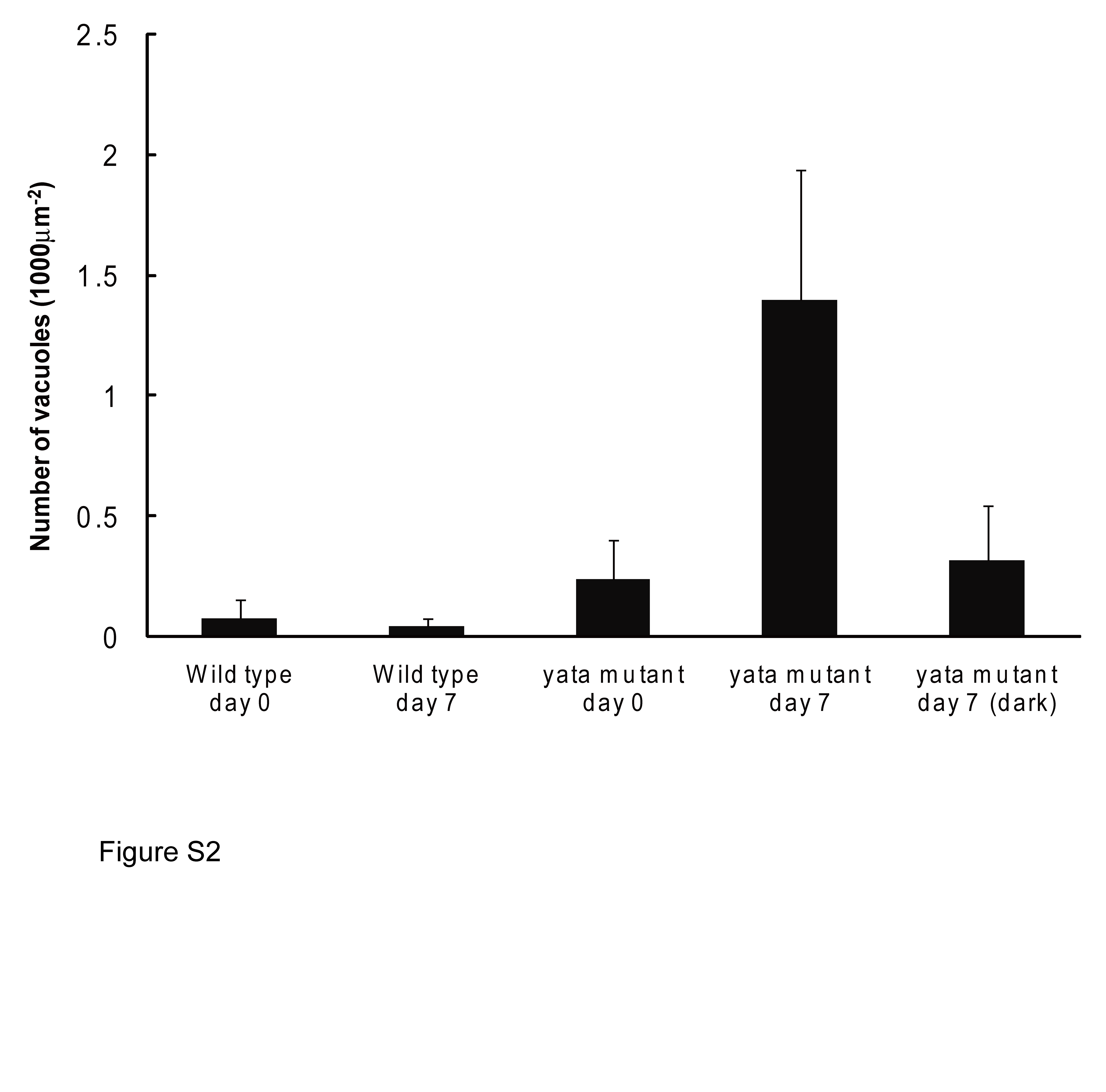

Supplement: Figure S2 — Measurement of vacuolization in the compound eye. The number of vacuoles with a longest diameter of over 5 µm was measured in the compound eyes of different genotypes, of different ages and under different light-conditions. An increased number of vacuoles was observed in day 7 yata mutants, although this number was suppressed in constant dark conditions. The numbers of the examined samples were 2 (w, day0), 4 (w, day7), 2 (w; yataKE2.1/yataKE2.1, day0), 5 (w; yataKE2.1/yataKE2.1, day7), 4 (w; yataKE2.1/yataKE2.1, day7, constant dark), (2.52 MB TIF) [file pone.0004466.s002.tif]

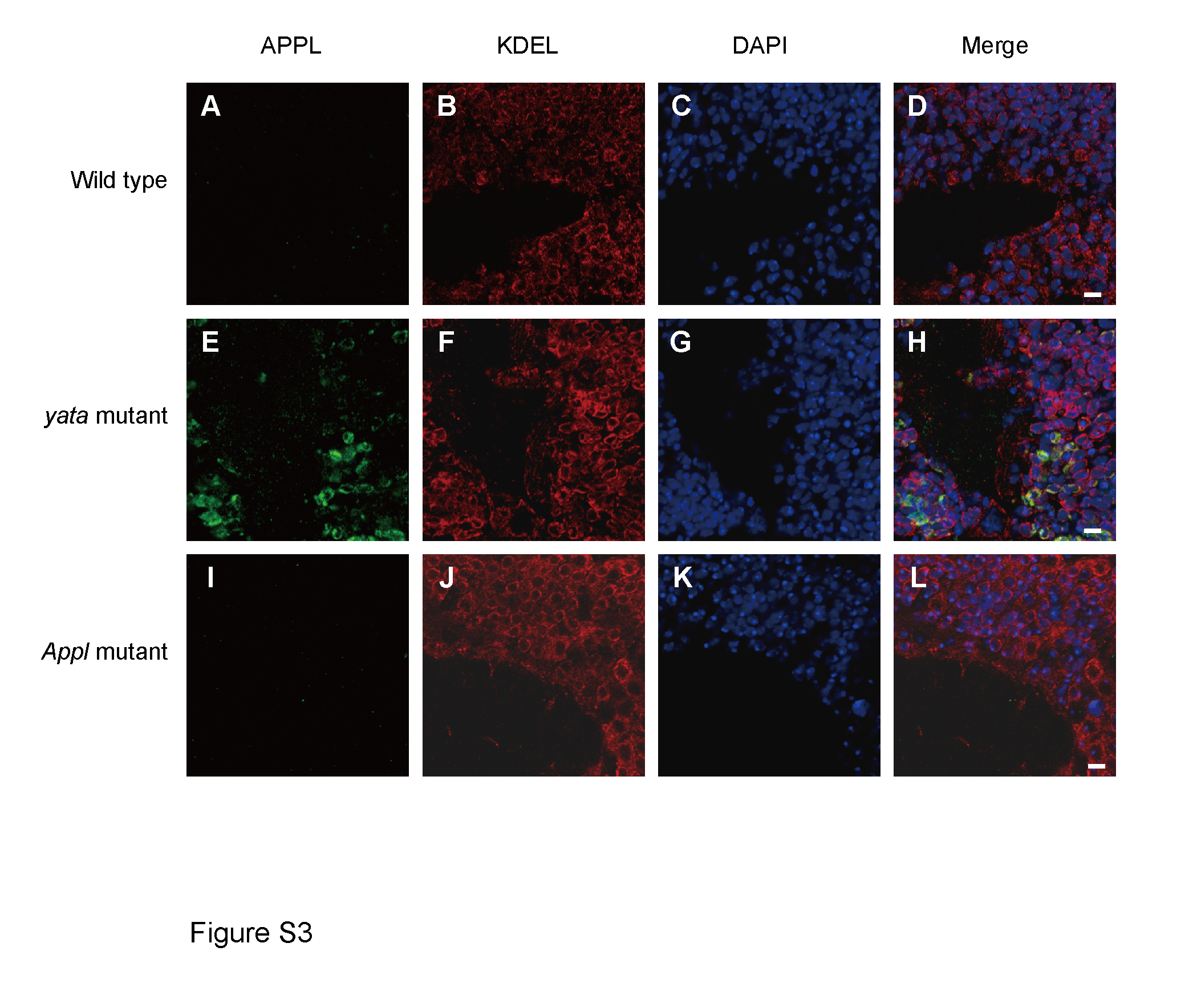

Supplement: Figure S3 — Confocal microscopic analyses of APPL localization. Localization of endogenous APPL in the wild-type, yata null mutant and Appl null mutant pupae. The pupae were examined and observed under identical conditions. Immunoreactivity of APPL was observed to accumulate in cell bodies of yata mutant pupa (E), but not in wild-type (A) and Appl mutant pupae (I). Staining with the anti-KDEL antibody (B, F, J), which labels the ER; DAPI, which labels nuclei (C, G, K); and merged images (D, H, L) are also shown. Scale bars: 5 µm. (2.57 MB TIF) [file pone.0004466.s003.tif]

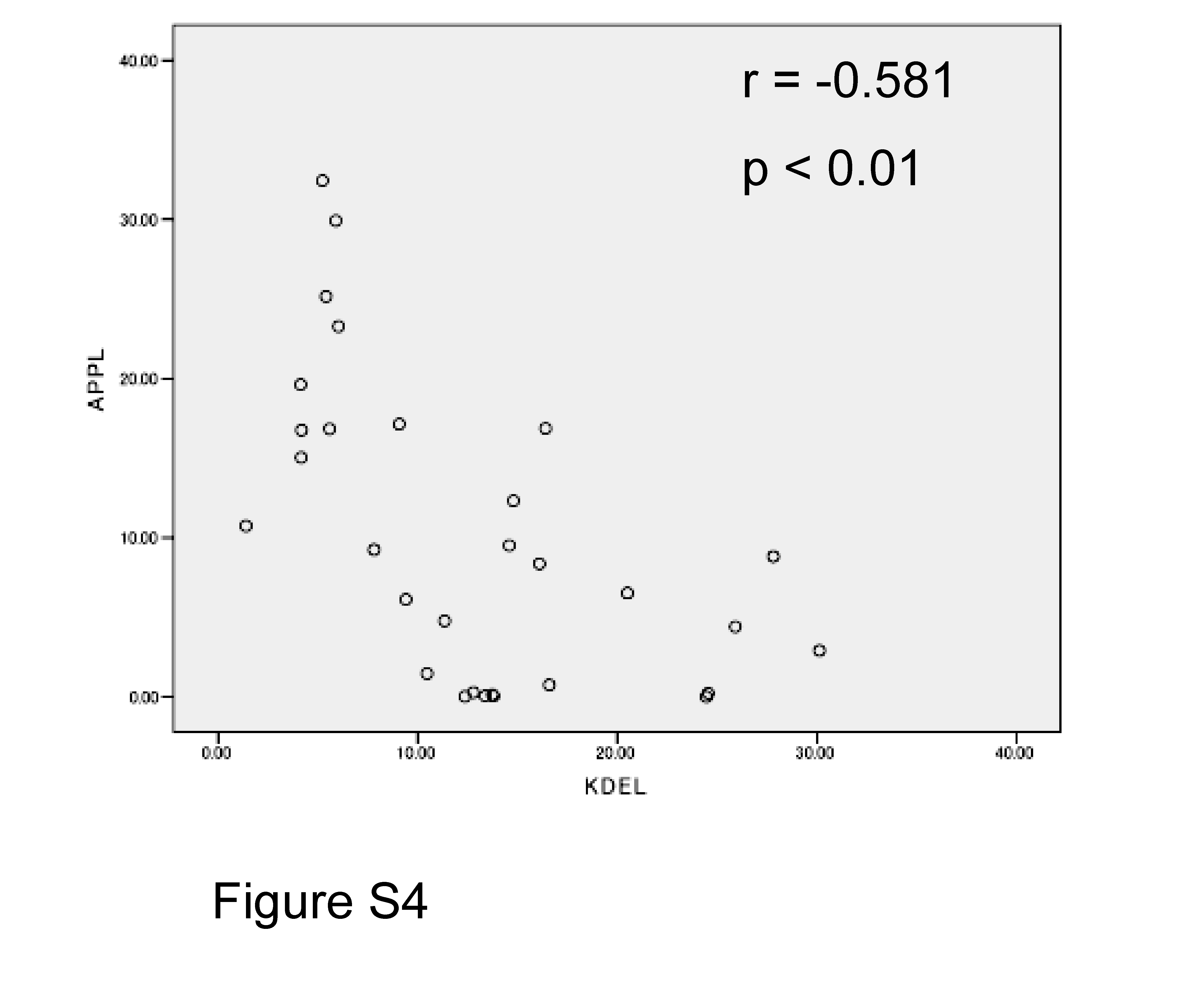

Supplement: Figure S4 — Correlation of the signal intensities of APPL and KDEL immunostaining. Statistical analysis of the immunostaining intensities of APPL and KDEL in the 10 µm square regions of the single optic lobe. A significant correlation was observed between the staining intensities of these two antibodies (r = −0.581, Pearson's correlation coefficient, p<0.01, N = 30). (0.71 MB TIF) [file pone.0004466.s004.tif]

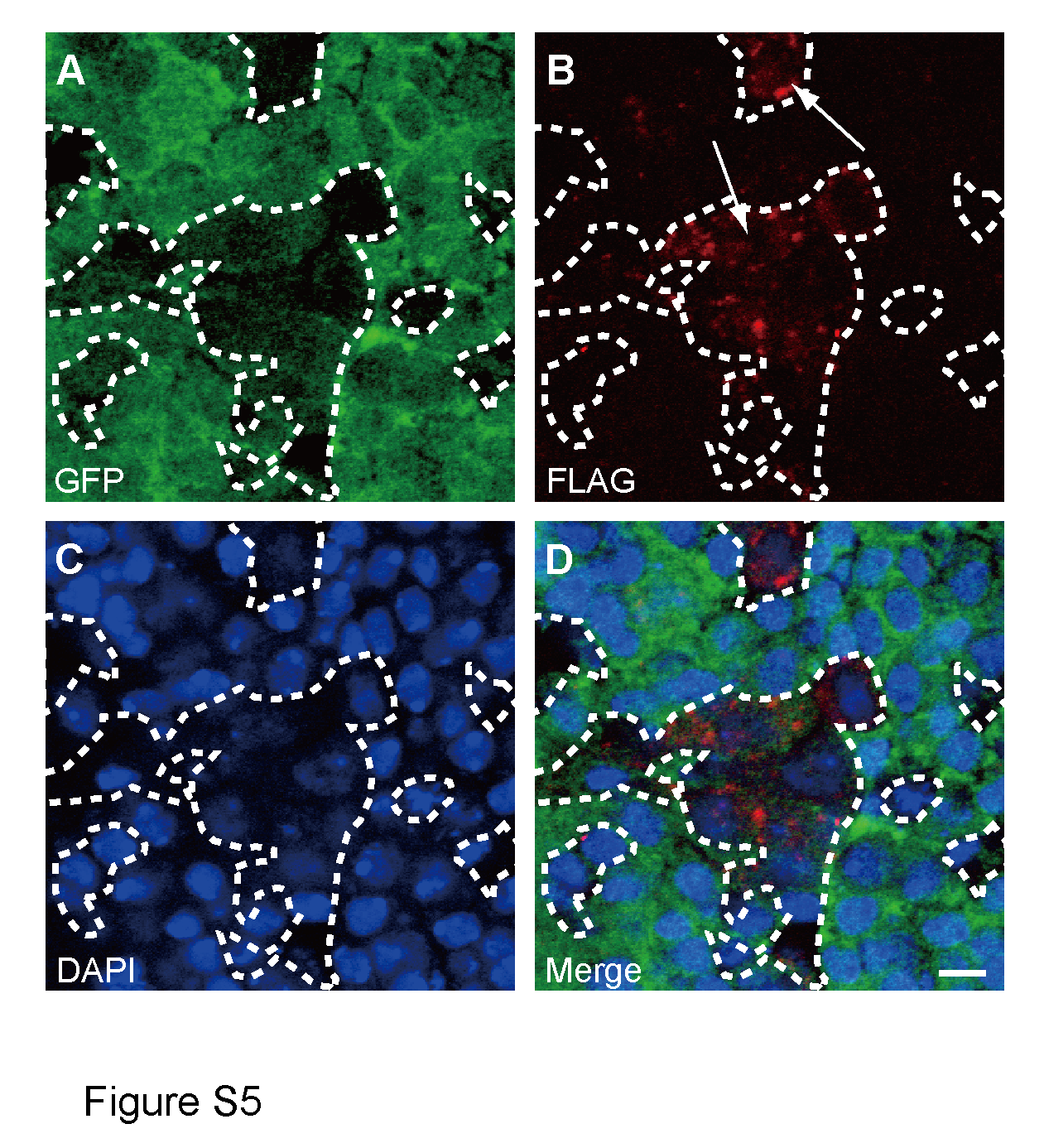

Supplement: Figure S5 — Genetic mosaic analyses of the subcellular localization of exogenously expressed APPL-FLAG. Accumulation of APPL-FLAG was observed in the yata mutant clones (A–D, surrounded by dotted lines, arrows in B), which were marked by the absence of GFP (A). Scale bar: 5 µm. (2.26 MB TIF) [file pone.0004466.s005.tif]

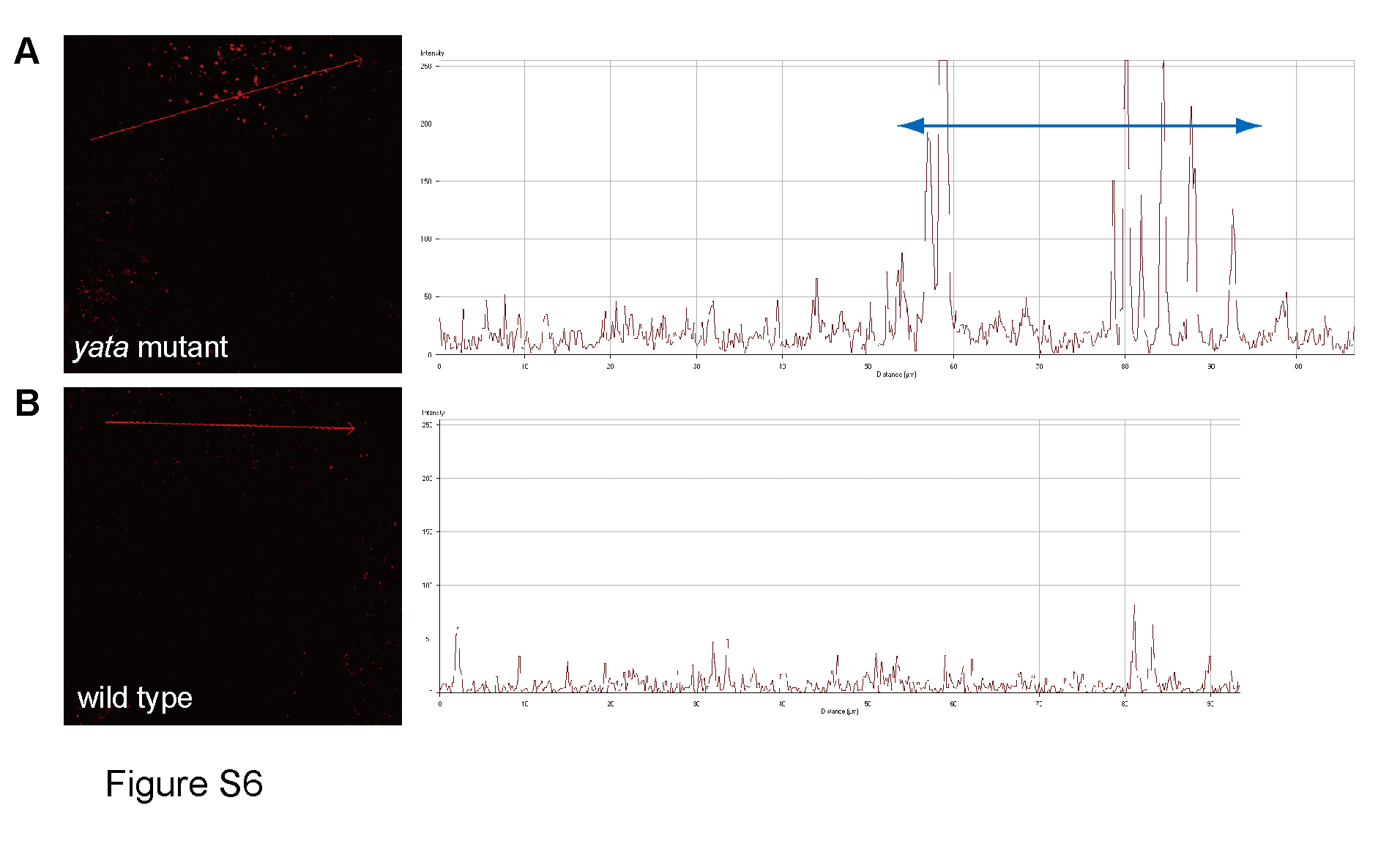

Supplement: Figure S6 — Profiles of Sec23p immunostaining. (A) Intensity profiles of Sec23p immunostaining of the yata mutant shown in Figure 7A. Radically elevated Sec23p signals are shown in the indicated region (blue arrow). (B) Such strong signals were not observed in the wild-type pupae, see Figure 7H. (0.37 MB TIF) [file pone.0004466.s006.tif]
